# Betel quid dependence is associated with functional connectivity changes of the anterior cingulate cortex: a resting-state fMRI study

**DOI:** 10.1186/s12967-016-0784-1

**Published:** 2016-02-02

**Authors:** Tao Liu, Jianjun Li, Zhongyan Zhao, Yuan Zhong, Zhiqiang Zhang, Qiang Xu, Guoshuai Yang, Guangming Lu, Suyue Pan, Feng Chen

**Affiliations:** Department of Neurology, Nanfang Hospital, Southern Medical University, 510515 Guangzhou, China; Department of Neurology, People’s Hospital of Hainan Province, 570311 Haikou, China; Department of Radiology, People’s Hospital of Hainan Province, Xiuhua Road 19, Xiuying District, 570311 Haikou, China; Department of Medical Imaging, Jinling Hospital, Clinical School of Southern Medical University, 210000 Nanjing, China; School of Psychology, Nanjing Normal University, 210000 Nanjing, China

**Keywords:** Betel quid, Drug dependence, Resting-state fMRI, Resting-state functional connectivity

## Abstract

**Objective:**

It is generally acknowledged that drug dependence is connected with abnormal functional organization in the individual’s brain. The present study aimed to identify the anterior cingulate cortex (ACC) abnormality with the cerebral networks involved in betel quid dependence (BQD) by resting-state functional connectivity (rsFC) using functional magnetic resonance imaging (fMRI).

**Methods:**

With fMRI data measured from 33 resting-state BQD individuals and 32 non-addicted and age-, sex-, education-matched healthy controls, we inquired into the BQD-related changes in FC between the regions of ACC with the whole brain involved in BQD individuals using a region of interest vised method, and to identify the relation of the alteration with the severity of BQD and duration.

**Results:**

Compared to controls, the BQD group showed increased connectivity from ACC to the regions of the reward network (brainstem including midbrain regions such as the ventral tegmental area and pons, caudate, thalamus) and cerebellum. Decreased connectivity was observed in the BQD group in regions from ACC to the default mode network (medial prefrontal cortex and precuneus) and para Hippocampal/hypothalamus. Specifically, the BQD scale was positively correlated with increased FC of right ACC to left thalamus and left ACC to pons; the durations were negatively correlated with FC of right ACC to left precuneus.

**Conclusion:**

These disturbances in rsFC from ACC to the reward network and DMN revealed by fMRI may have a key function in providing insights into the neurological pathophysiology underlying BQD-associated executive dysfunction and disinhibition. These findings may contribute to our better understanding of the mechanisms underlying BQD.

## Background

In Hainan province of China, Betel quid (BQ) is the combination of fresh Areca nut (AN) and slaked lime (i.e. aqueous calcium hydroxide paste) wrapped in a betel leaf without tobacco and other ingredients. BQ is chewed by approximately 600 million people globally, with its use concentrated in South Asia, Southeast Asia, and Pacific islands [1]. BQ chewing is an important behavior from a public health perspective because it is associated with a variety of health issues, most notably oral cancer and precancerous conditions such as leukoplakia and oral submucous fibrosis [2]. Consequently, BQ has been categorized into a Group 1 carcinogen by the International Agency for Research on Cancer [1, 3]. BQ ranks the fourth most frequently consumed psychoactive substance around the globe, following only alcohol, nicotine, and caffeine in prevalence [4]. BQ use is related to a dependency syndrome, which is characterized by enhanced concentration, mild euphoria, relaxation, postprandial satisfaction and a withdrawal syndrome involved with sleeplessness, unstable mood, irritability and anxiety, and its severity can be similar to that of amphetamine [5]. However, the mechanism by which the BQ dependence (BQD) behaviour influences cerebral function through working on the specific cerebral areas has not yet been clearly illuminated. Our previous voxel based morphometry (VBM) study of BQD indicated that BQD individuals have gray matter volumes decreased in the right rostral anterior cingulate cortex (ACC), which also indicated a negative correlation with the duration of BQD [6]. These findings might be helpful to investigate potential structural substrates of the BQD.

The use of chronic addictive drug is usually associated with abnormal functional organization in the user’s brain, which results in executive dysfunction [7] and disinhibition [8] to the drug and drug-related cues and makes sure their compulsive patterns of drug-seeking actions [9]. The connection of spontaneous fluctuations of blood oxygen level-dependent signals in diverse areas of the “resting” brain assesses the resting-state functional connectivity (rsFC) which is considered as a measure of its functional organization [10]. Studies have outlined a number of resting-state networks corresponding to key brain functional organizations including vision, movement, language, audition, executive function, episodic memory, and salience detection [10]. It is thought that the default mode network (DMN) [11], the most famous resting-state network consisting of brain regions deactivated during external-oriented tasks, is involved in the maintenance of the baseline brain activities related to cognitions of self-awareness, episodic memory and interactive modulation between the interior mind activities and exterior tasks [12]. The sensorimotor network (SMN) [13], which is significant for the performance of voluntary movements, functionally connects regions within the primary motor cortex (M1) and the supplementary motor area (SMA) [14, 15]. The affective network (AN) comprises a corticolimbic circuit responsible for negative emotional arousal or regulation [16–18] and participates in autonomic and visceral functions [19, 20]. Respectively, the cognitive network (CN) and the visual network (VN) cope with corresponding cognition and vision functions [21].

Since it is more readily applicable than functional activation MRI in clinical environment, several groups have started to analyze the rsFC in various neuropsychiatric diseases such as Alzheimer’s disease and depression [22, 23]. According to models of addiction, the major brain regions underlying addiction constitute a network of at least four interdependent and overlapping circuits [24]: (i) motivation and/or drive and salience evaluation, located in the orbital frontal cortex; (ii) cognitive control, located in the dorsal anterior cingulate cortex and prefrontal cortex; (iii) memory and learning, including the hippocampus and amygdala; and (iv) reward, involving the ventral pallidum and nucleus accumbens. In addition, the ACC is an essential part of the frontal-subcortical circuit, and it plays a modulatory role in cognition, emotion and reward expectations [16, 25–27], and is also likely to affect the reactivity of the above circuits and parts of this network [16, 28, 29]. Convergent evidence from lesion and neuroimaging studies consistently indicate that drug dependence is closely associated with structural [30], functional [31], and metabolic [32] abnormalities of the ACC. Recent molecular and neuroimaging studies have further theorized that altered baseline or resting-state activity in the ACC is associated with drug dependence [33–35].

To date, the rsFC within the key regions of BQD has not been extensively studied. Therefore, in this study we detected whether there was alteration of functional connectivity related to ACC on samples with BQD for investigating the mechanisms underlying BQD.

## Methods

### Ethics statement

This study was approved by our research ethics review board of the People’s hospital of Hainan Province, Haikou, China according to the Declaration of Helsinki (2000) (No. SYLL2012012). The consent form has been read and signed by each subject before being included in the study.

### Participants

Persons with exclusive use of BQs were included, the use of BQ without tobacco at least 1 day at a time for no less than 5 years were categorized as “individuals using BQ without tobacco.” Persons without use of BQ, areca nut and tobacco (in all forms) were defined as “healthy controls.”

Assessment of BQD has been generally conducted upon the Diagnostic and Statistical Manual of Mental Disorders-IV (DSM-IV, American Psychiatric Association, 2000), the International Classification of Diseases-10 (ICD-10, World Health Organization, 1992) [36–39] and some dependence scales for other substances such as opioids [40] or tobacco [41]. Recently, Lee [42] developed an initial instrument specially for measuring BQ dependence: the Betel Quid Dependence Scale (BQDS), which is more suitable for Chinese-speaking chewers and valid for current English-speaking male and female chewers in Guam [43]. The BQDS is comprised of three factors: “physical and psychological urgent need,” “increasing dose,” and “maladaptive use” [42, 43], which was found to have good internal consistency (α = 0.92) and construct validity [42]. During the first session we administered the BQD Scale (BQDS). BQD participants conformed to the criteria for present BQ addiction, as diagnosed by the total BQDS >4 [42]. Cross-sectional studies on samples of individuals had demonstrated high comorbidity of addiction with psychiatric disorders, particularly affective disorders (including depression), anxiety disorders (generalized anxiety disorder and social anxiety disorder). In order to eliminate the interference of depression and degrees of anxiety, all participants were evaluated with the self-rating anxiety scale (SAS) and self-rating depression scale(SDS)on the scanning day. The scores of SAS and SDS should be less than 50.

The exclusion criteria were as follows: (1) tobacco smokers; (2) persons with use of different forms of tobacco without smoke e.g. gutka and/or paan masala; (3) persons with self-claimed systemic diseases such as neurological disorder, cardiovascular disease, diabetes mellitus, epilepsy, thyroid and renal disorders; (4) persons with present or recent history of any Axis I psychiatric and/or substance use diseases; (5) present use of psychotropic drugs; (6) left-handers; and (7) not able to read and write Chinese.

A questionnaire in simple Chinese acquiring information including age and gender as well as monthly income, educational status, duration of BQ chewing habit, daily dosage of BQ and duration time of quid placement in mouth was distributed to all participants. The substance use diseases including alcohol use disorder have been ruled out prior to examination, but wine has played an important role in Chinese social aspects of life, all participants were also assessed for alcohol use in the past 30 days, including the average frequency of drinking and number of drinks per occasion. Alcohol consumption was recorded intactly according to the individuals’ reports. However, most individuals could only provide vague descriptions such as “one pack” or “half a pack” instead of stating exact quantity of daily consumption. So we had to transform such descriptions into figures, based on our knowledge of the commercial packs of each product. For instance, the alcohol is sold in small bottles (100 mL) and standard bottles (500 mL). We recorded alcoholic beverages as beer or white spirit (a Chinese distilled beverage with about 50 % alcohol content), which were considerably consumed in Mainland China. One gram pure ethanol would approximately equal to 18.3 cc of beer, or to 2 cc of white spirit.

At last, 38 BQD volunteers and 36 control individuals recruited from a residential area of Wanning City of Hainan province, China were included.

### MRI data acquisition

MRI data were obtained on a Siemens Verio3T MRI scanner using a standard 6-channel head coil (Erlangen, Germany) in the Department of Radiology, People’s hospital of Hainan Province. During the scanning, subjects were required to remain their eyes closed and at the same time stay awake, to insure thinking of nothing particularly, and to maintain their heads still. In order to rule out gross cerebral pathology, a routine structure MR scan was conducted. Then spin-echo imaging was used to collect anatomical images of the functional slice locations in the axial plan parallel to the Anterior Commissure-Posterior Commissure (AC-PC) line. Whole-brain functional images were acquired with a T2*-weighted EPI sequence sensitive to BOLD contrast (repetition time = 2000 ms, echo time = 30 ms, field of view = 240 × 240 mm, flip angle = 80°, image matrix = 64 × 64, voxel size = 3.75 × 3.75 × 5 mm. Each brain volume included 31 axial slices and each functional run comprised 240 volumes). A high-resolution T1-weighted structural image was acquired using a MPRAGE sequence (repetition time = 2300 ms, echo time = 2.9 ms, TI = 900 ms, field of view = 256 × 256 mm, flip angle = 9°, in-plane matrix = 256 × 256, slice thickness = 1 mm, no gap, and voxel dimension = 1 × 1 × 1.33 mm).

### MRI data preprocessing

The toolbox Data Processing Assistant for Resting-State functional MR imaging (DPARSF; http://www.restfmri.net/forum/DPARSF) [44] were used for the preprocessing of fMRI imaging data through statistical parametric mapping (SPM8; http://www.fil.ion.ucl.ac.uk/spm/) and an rs-fMRI data analysis toolkit (REST1.8; http://www.restfmri.net). The first 10 volumes of each functional time series were removed for the magnetization equilibrium. Slice timing and realignment for head motion correction were performed. Spatial normalization to the standard Montreal Neurological Institute (MNI) echo-planar imaging template in the Statistical Parametric Mapping package; We then spatially normalized the functional images to standard coordinates and resampled to 3 × 3 × 3 mm [45]. All subjects with head motion >1.5 mm translation or >1.5° rotation in all directions were ruled out. Lastly, we smoothed the resampled images with a Gaussian kernel of 4 mm. Then we performed linear trend and band-pass filtering (0.01–0.08 Hz) for the purpose of removing the influence of low-frequency drift and high-frequency noise.

### Definition of seed regions

WFU PickAtlas Tool Version 3.0 (http://fmri.wfubmc.edu/software/PickAtlas) was adopted to define the seed region of bilateral ACC. It was a toolbox which offered a method of generating regions of interest (ROIs) based on atlas [46]. The automated anatomical labeling (AAL) atlas offered in this toolbox was adopted to define the ROI of bilateral ACC which was used to define the reference time series with the method discussed in previous rs-fMRI studies [47].

### Functional connectivity analyses

Functional connectivity analyses were carried out using the REST software. The reference time course was acquired by computing the mean time series for ROI. Cross-correlation was then analyzed between the mean signal change in the ACC and the time series of every voxel of whole brain. Lastly, we applied a Fisher Z-transform for the purpose of improving the normality of the correlation coefficients [48]. In order to rule out possible effects of factors such as global, WM, and CSF signals, six head motion parameters and their mean time series were included in the regression analysis.

### Statistical analysis

SPSS software (version 16.0; SPSS, Inc., Chicago, IL) was used to compare the demographic and clinical variables between the two groups. We performed an independent two-sample *t* test for continuous variables, and a Chi square test for proportions. *P* values <0.01 were regarded as statistically significant.

Within each group, a random effect one-sample *t* test was performed on an individual Z value map in a voxel-wise manner to determine brain regions showing significant functional connectivity to the seed region of bilateral ACC. Significant thresholds were set at a corrected *P* < 0.05 with multiple sample correction using false discovery rate (FDR) criterion [49] across the whole brain.

To identify regions with a significant difference in connectivity to the bilateral ACC in a voxel-wise manner, we entered these individual Z values into the SPM8 software for random-effects analysis two-sample *t* tests. For the purpose of controlling for the possible influences of factors including age, sex and education years on the results, such factors were included as nuisance covariates. We set the thresholds at a corrected *P* < 0.01, with multiple comparisons correction using the Alpha Sim program determined by Monte Carlo simulation (*P* < 0.05, a minimum 22 clusters).

Correlation analysis between the BQDS,duration and the fMRI data was accomplished. Firstly, we extracted clusters that had significant differences in ACC functional connectivity between groups. Secondly, we calculated the mean Z values of abnormal functional connectivity region mask within every BQD individuals. Finally, we used SPSS for Windows (version 16.0, Chicago, IL) to analyze the Pearson correlation coefficients between abnormal Z values and BQDS. *P* < 0.05 was regarded to have statistical significance.

## Results

38 BQD individuals and 36 healthy control subjects were recruited for this study. 3 BQD individuals with angio cavernoma, arachnoid cyst and lacunar infarction respectively were excluded for lesions of the brain. Two BQD individuals and four healthy control subjects were excluded because of excessive head movement.

### Demographics and clinical characteristics

Sixty-five subjects (33 BQD users and 32 controls) were selected to be analyzed in the final data. The BQD individuals and control groups did not differ in terms of age, sex, or education. In order to eliminate the interference of depression and degrees of anxiety, all participants were evaluated with the SAS and SDS, and the results failed to reach cut-off for clinical significance on average although the SDS scores in BQD group were significantly lower than those in controls. The dosages of alcohol in the past 30 days in BQD and HC group, were 200.2 ± 34.8 g and 189.0 ± 33.4 g respectively, which indicated they were moderate users [50], combined with no alcohol use disorder. We speculated that there were no alcohol caused effects on BQD use. No significant differences were observed in monthly income, alcohol use during the 30 days prior to the examination and SAS between the two groups (*P* values >0.05). Individuals indicated that they had been chewing BQ with dependency syndrome for a mean duration of 20.6 ± 6.9 years (range 7–31 years), a mean BQDS of 10 ± 3.4 (range 5–16) and consumed an average of 342 ± 106 g/day BQ (range 200–500 g/day) daily. BQD chewers placed BQ in their mouth for an average of 7.6 ± 2.4 min (range 3–12) before spitting-out the remnants. However, the BQD individuals had a lower SDS score than HC (*P* values <0.05). Table 1 summarizes the demographics of BQD and Healthy control participants.

**Table 1:** Demographics and clinical characteristics of participants

	Age	Sex (male/female)	Education (years)	Monthly income (US $)	Duration of placement of BQ in the mouth (min)	Dosage of BQ (g/day)	Duration of BQ (years)	BQDS	Alcohol last 30 days (g)	SAS	SDS
BQD	46.7 ± 9.4	24/9	12.3 ± 2.7	423.5 ± 73.7	7.6 ± 2.4	342 ± 106	20.6 ± 6.9	10 ± 3.4	200.2 ± 34.8	27.2 ± 5.6	28.6 ± 6.6
HC	45.8 ± 9.3	20/12	12.6 ± 2.4	413.1 ± 73.0	N/A	N/A	N/A	N/A	189.0 ± 33.4	28.3 ± 6.1	32.8 ± 7.5
Statistics	0.446	0.777	0.512	0.799					1.025	0.759	2.385
*P* value	0.657	0.378	0.610	0.427					0.309	0.451	0.020

Unless otherwise indicated, data are means ± SD. The *P* value for gender distribution in the two groups was obtained by Chi square test. The *P* value for age, monthly income, education, alcohol last 30 days, SAS, SDS difference between the two groups was obtained by independent-samples *t* test

*N/A* not applicable, *BQ* betel quid, *BQD* betel quid dependence, *HC* healthy control, *BQDS* betel quid dependence scale, *SAS* self-rating anxiety scale, *SDS* self-rating depression scale

### Within-group comparison of the ACC resting-state functional network

Correlation maps were produced by extracting the BOLD time course from bilateral ACC then computing the correlation coefficient between that time course and the time course from all other brain voxels. Positive z-score values are significantly correlated or positive with the seed region, whereas negative z-score values are significantly anti correlated or negative [51].

**Fig. 1:**
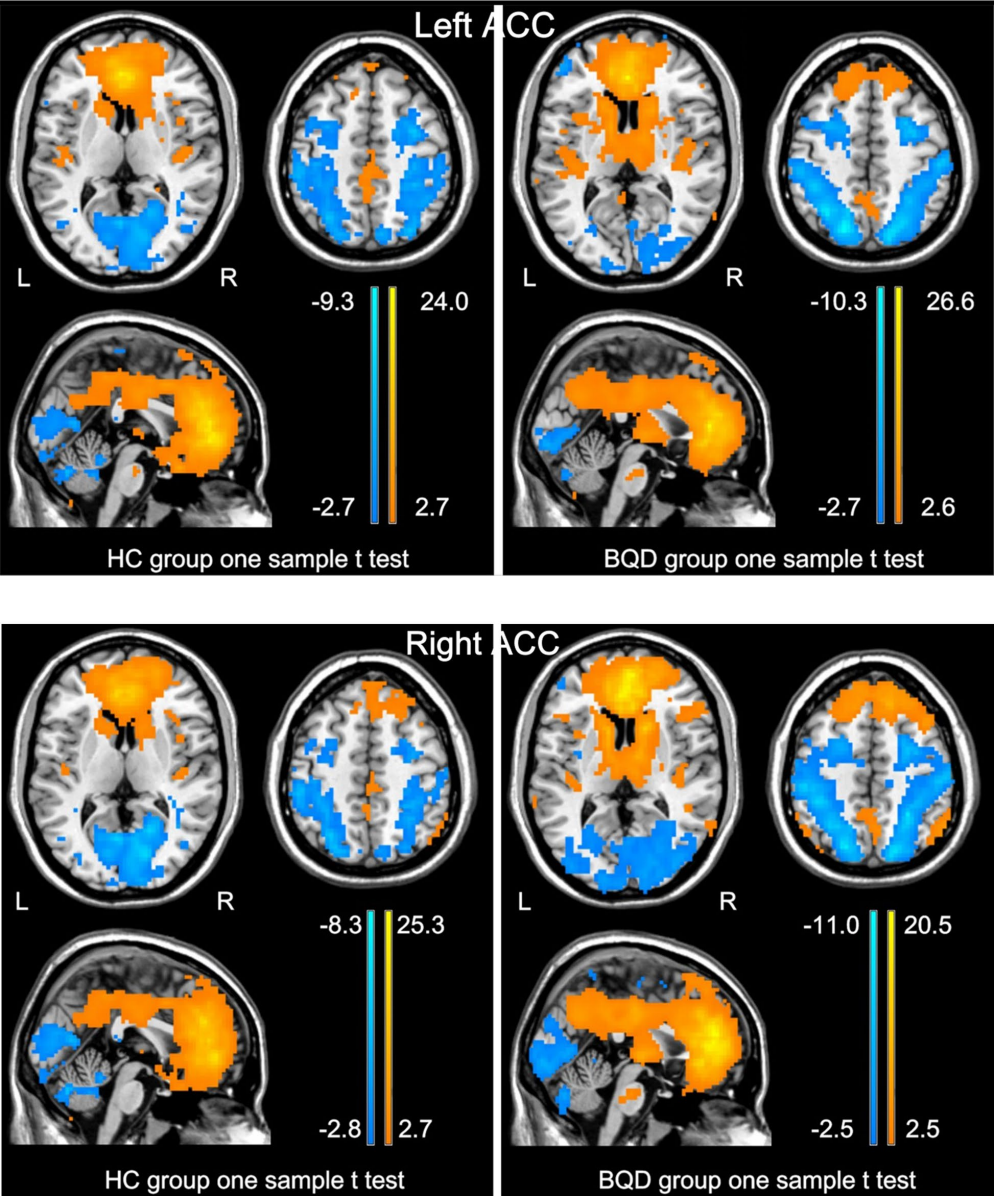
One sample *t* test result of bilateral ACC functional connectivity for BQD and HC group. In all subjects, the ACC positively correlates with the PCC/precuneus, lateral parietal cortex, medial prefrontal cortex, superior frontal gyrus, and the middle and inferior temporal gyri, the medial prefrontal cortex, orbitofrontal cortex, temporal lobe, insula, thalamus, caudate, putamen, and other cingulate subregions (*P* < 0.05, FDR corrected). The ACC shows negative functional connectivity with the precentral and postcentral gyri, prefrontal cortex, parietal cortex, cuneus lobe, lingual and fusiform gyri (*P* < 0.05, FDR corrected). *ACC* anterior cingulate cortex, *PCC* posterior cingulate cortex, *HC* healthy control, *BQD* betel quid dependence

The results from the one-sample *t* test showed the spatial distribution of functional connectivity patterns of bilateral ACC with the whole brain for all subjects were similar, although potentially interesting variations can be seen (Fig. 1). The functional connectivity patterns of ACC were also very similar in the right and left hemispheres. In all subjects, the positive functional connectivity with ACC (orange) mainly involved (i) the DMN, including medial prefrontal cortex, posterior cingulate cortex (PCC)/precuneus, the middle and inferior temporal gyri (MTG/ITG), and superior frontal gyrus; (ii) the AN covered the orbitofrontal cortex, medial prefrontal cortex, and temporal pole, the thalamus, insula, putamen and caudate (*P* < 0.05, FDR corrected) (Fig. 1). On the other hand, the negative functional connectivity with the seed regions (blue) mainly involved (i) the SMN, which included the precentral and postcentral gyri; (ii) the CN, which included the dorsolateral prefrontal cortex (dlPFC), ventrolateral prefrontal cortex (vlPFC) and dorsolateral parietal cortex; and (iii) the VN, which included the cuneus lobe, and lingual and fusiform gyri (*P* < 0.05, FDR corrected) (Fig. 1).

### Inter-group comparisons of the ACC resting-state functional network

**Fig. 2:**
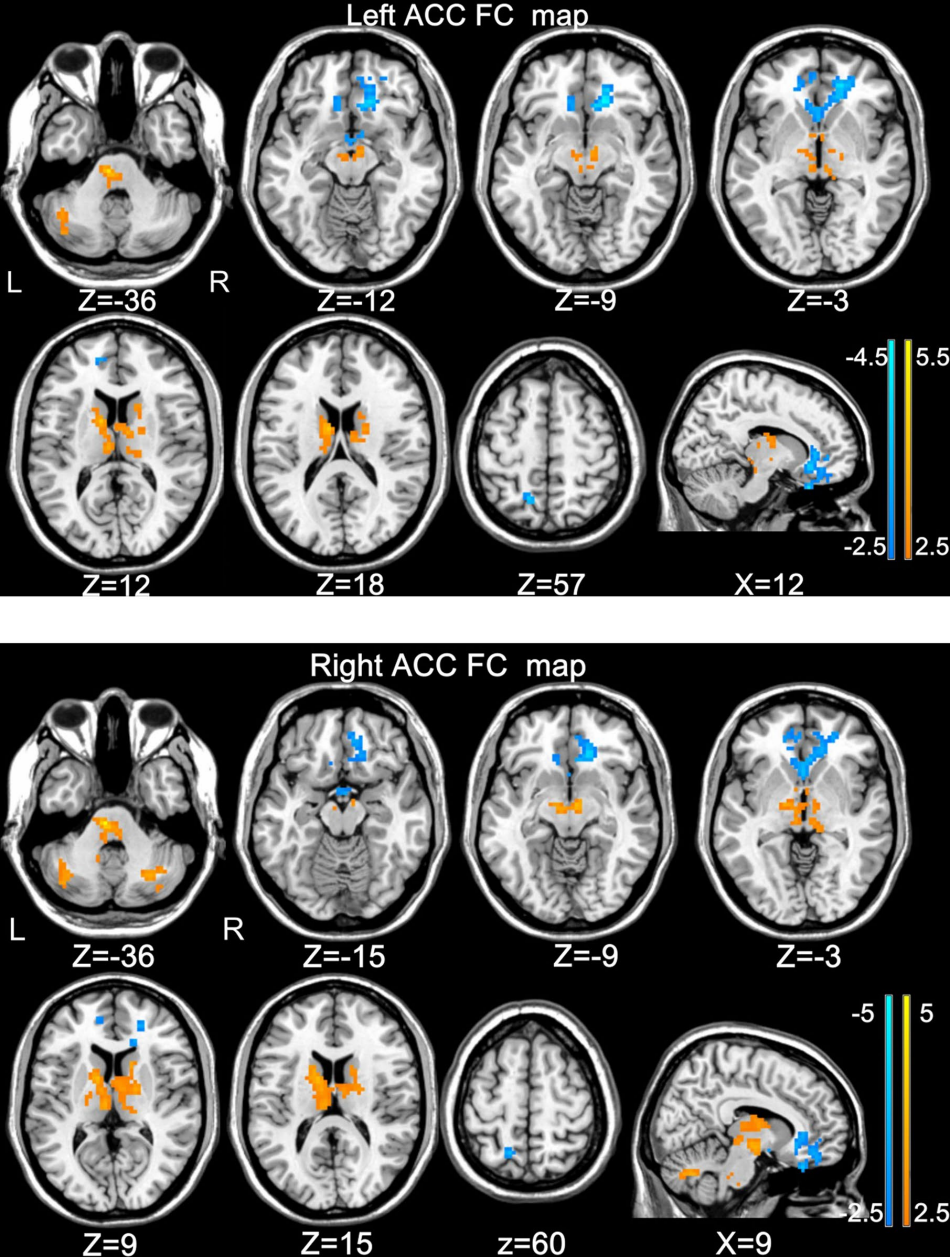
Significant differences in the functional connectivity from the left ACC and right ACC to whole brain between BQD individuals and healthy controls. Thresholds were set at a corrected *P* < 0.01, determined by Monte Carlo simulation. For display purposes only, all statistical maps are overlaid on a T1-weighted MNI template using MRIcron (*ACC* anterior cingulate cortex, *BQD* betel quid dependence, *MNI* montreal neurological institute)

Functional connectivity differences between HCs and BQD from two-sample *t* tests, are displayed in Table 2 and Fig. 2 (Alpha Sim corrected, *P* < 0.01).The individuals with BQD indicated significant increases in the FC (orange) between bilateral ACC and pons, between bilateral ACC and caudate, between bilateral ACC and bilateral thalamus, between bilateral ACC and midbrain, between bilateral ACC and cerebellum (Fig. 2). By contrast, bilateral ACC exhibited decreased functional connectivity (blue) to the medial prefrontal cortex (mPFC), the left precuneus, and the left para Hippocampal/hypothalamus (Fig. 2). Critically, the right ACC maps are to a large extent sign-inverted versions of the left ACC maps (Fig. 2).

**Table 2:** Abnormal functional connectivity of bilateral ACC in BQD individuals compared with healthy controls

Brain region	BA	Voxels	Maximal *t* value	MNI coordinates		
				X	Y	Z
Left ACC						
Pons		61	5.23	−6	−27	−33
L. caudate		38	4.26	−12	3	12
L. thalamus		53	3.44	−6	−18	12
R. thalamus		51	3.37	9	−21	9
Midbrain		43	2.98	−6	−12	−12
Cerebellum posterior lobe		27	3.10	−42	−57	−36
vmPFC, ACC	47	424	−4.5	15	30	−9
L. precuneus	7	32	−3.68	−15	−57	57
L. parahippocampal/hypothalamus		33	−3.36	−12	0	−18
Right ACC						
Pons		114	5.87	−6	−24	−36
L. caudate		43	4.90	−12	3	12
R. caudate		41	3.45	15	15	9
L. thalamus		117	3.95	−6	−18	12
R. thalamus		64	3.91	18	−9	6
Midbrain		88	4.18	6	−9	−9
L. cerebellum posterior lobe		49	4.07	−36	−66	−33
Cerebellum anterior lobe		62	3.87	6	−60	−30
R. cerebellum posterior lobe		53	3.73	30	−66	−36
mPFC, ACC	32.47	461	−4.47	18	27	−27
L. precuneus	7	24	−3.80	−15	−57	60
hypothalamus/parahippocampal_L		24	−3.35	3	0	−15

A corrected threshold of *P* < 0.01 determined by Monte Carlo simulation was taken as meaning that there was a significant difference between groups

*L* left, *R* right, *mPFC* medial prefrontal cortex, *ACC* anterior cingulate cortex, *BA* Brodmann’s area, *MNI* montreal neurological institute, *x, y, z* coordinates of primary peak locations in the MNI space

### Correlation analysis results

**Fig. 3:**
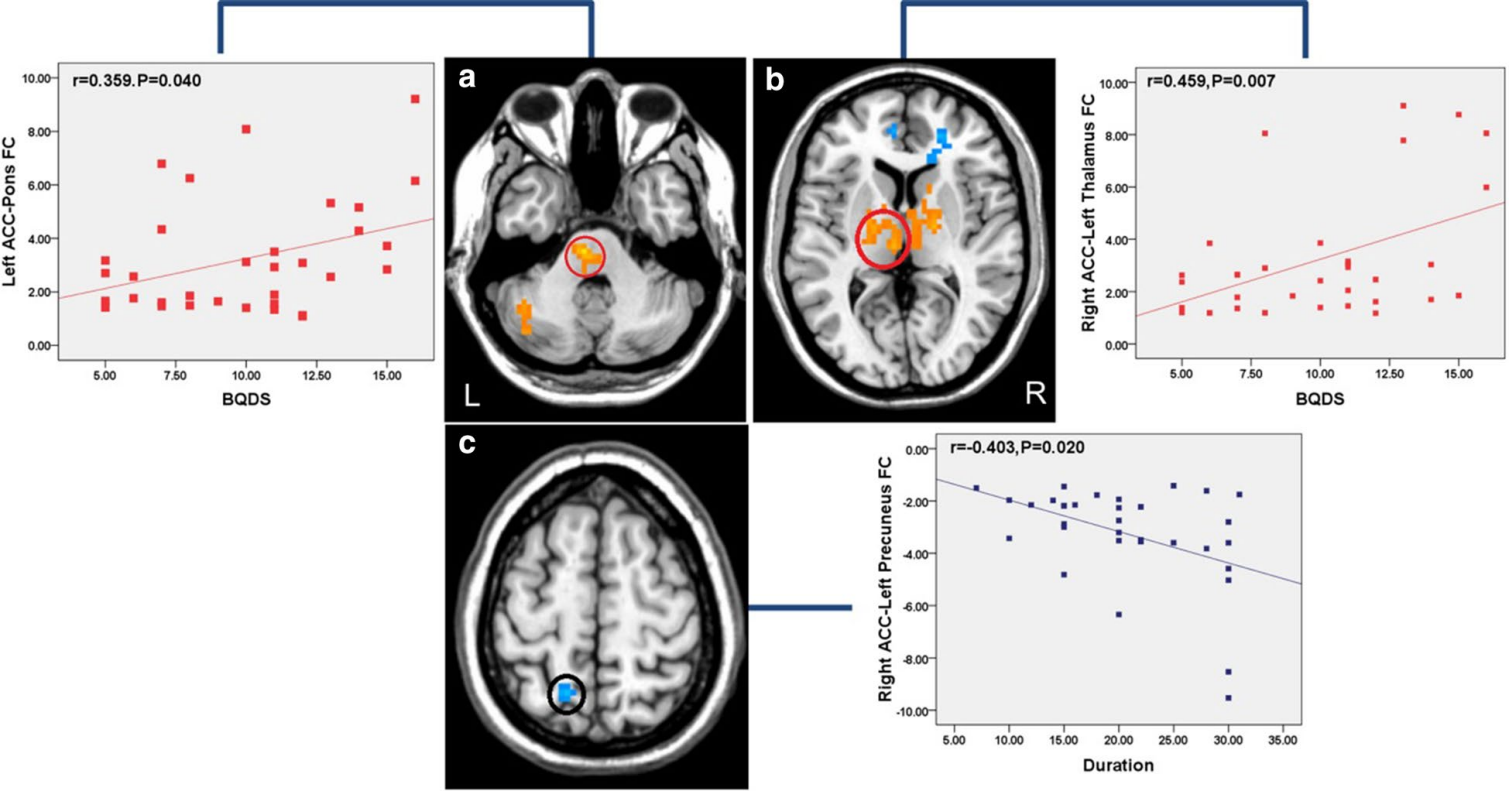
Significant correlations between the functional connectivity and clinical variables. **a** Correlation between BQDS and the functional connectivity of left ACC-pons (*r* = 0.359; *P* = 0.040). **b** Correlation between BQDS and the functional connectivity of right ACC-left thalamus (*r* = 0.459; *P* = 0.007). **c** Correlation between duration and the functional connectivity of right ACC-left precuneus (*r* = −0.403; *P* = 0.020)

In BQD individuals, the functional connectivity of right ACC to left thalamus and left ACC to the pons were significantly and specifically positively correlated with BQDS (*r* = 0.459; *P* = 0.007; *r* = 0.359; *P* = 0.040) (Fig. 3). In addition, the functional connectivity of right ACC to the left precuneus were negatively associated with durations (*r* = −0.403, *P* = 0.020) (Fig. 3). The other increased or decreased functional connectivity to the ACC were separated from BQDS and durations.

## Discussion

In the current rsFC study, we investigated the potential alterations of functional connectivity related to ACC on BQD and the correlation of functional connectivity changes with the severity and duration of BQD. Interestingly, we found the number of male individuals were higher than that of female individuals in BQD group. The functional connectivity results show that the aberrant functional connectivity during resting state between ACC and regions of reward and cognitive control network, which is associated with the neural mechanism of BQD. It has been reported that the cause of addiction is not onefold—including genetic—but that genes do occupy a vital position “by influencing processes in the brain and body [52–54] that interact with one another and with an individual’s life experiences to produce susceptibility or protection “[55], so the sex difference and structural plasticity in vulnerability to addiction may be implicated in those genetic backgrounds. These, of course, need further researches.

In this study, the ACC functional connectivity patterns mapped in HCs, and BQD individuals were in line with those reported in the previous study [56]. The ACC functional connectivity positively correlated with the DMN, AN, and brain regions that process different aspects of emotionally salient stimuli, the thalamus, caudate, and putamen. The ACC functional connectivity negatively correlated with the SMN, CN, VN. All subjects had the similar functional connectivity patterns of the ACC. However, BQD individuals had intensity distribution difference in ACC functional connectivity patterns. This suggests that our findings on the ACC functional connectivity are reliable.

The results of rsFC by two-sample *t* tests, measured in whole brain with seeds in the bilateral ACC, indicate that dysfunctional integrations take place in the brains of resting-state BQD individuals. Compared to the HC group, the BQD group demonstrated greater connectivity from ACC to the regions of the reward network (midbrain regions, pons, caudate, thalamus). Specifically, the BQDS were positively correlated with increased functional connectivity of right ACC to left thalamus and left ACC to pons. The reward circuit, a core component for the development and monitoring of motivated behaviors, is now regarded to be embedded within the cortico-basal ganglia network. The role of the ACC has led to its inclusion in many major theories of addiction, where it is believed to form part of an inhibitory system that exercises control over reward-related behavior [57, 58]. Recent research has indicated that the midbrain and striatal areas which are involved in the reward remain more extensive than previous estimates. An increasing number of fMRI researches have started to study reward-processing midbrain/pons activity in individuals [59]. Midbrain dopamine (DA) neurons play a central role in a wide range of behaviors, from attention and motivation to motor control and reinforcement. Increased midbrain activation during anticipation of pleasant tastes [60, 61], anticipation of monetary gains [62], and during exposure to visual stimuli that evoke romantic love [63] have been reported by task-based fMRI researchers. The dorsal striatal circuitry plays a pivotal role during the development of habitual compulsive drug dependence [64]. Brain imaging research indicated that dopamine increases in the dorsal striatum (caudate and putamen) were induced by drug-associated cues, which was an effect that related to self-reports of craving [65, 66]. Previous study has not covered the thalamus extensively in terms of addiction. Nevertheless, this area has been increasingly implicated during addiction due to its integrative function in the regulation of arousal and attentional modulation. For instance, dopamine neurotransmission in the thalamus was increased with intravenous administration of a stimulant drug in cocaine users rather than in controls, which was an effect related to craving [67]. Both PET and fMRI results demonstrate that thalamic activation can be increased by primary and secondary rewards (vs. non rewards) [68]. The ACC-thalamus functional connectivity were increased compared with HC; together with the finding that the right ACC-left thalamus positively correlated with BQDS, indicated the thalamus can serve as a specific cerebral area during the BQD development. Based on the previous studies and our results above, we argue that the functional connectivity in ACC to the reward network may be correlated with cognitive management and behavioral dysfunctions directed by goal in BQ addiction at least in some degree [69, 70]. Although BQ is a weakly addictive substance, our outcomes suggested that which shares similar functional organization to other addictions related to substance, indicating a more general pathomechanism for diseases correlated with increased BQD.

Interestingly, we found increased functional connectivity between ACC with cerebellum in BDQ group. It was observed in Previous PET and fMRI studies that drug-conditioned cue elicited the increased metabolism and activation of cerebellum [71, 72]. Glucose metabolism was greatly increased in the cerebellum when addicts performed reward expectation tasks [73], which suggested that the cerebellum is also included in drug-conditioned memories in addicts. And the cerebellum has been noted and described to play a compensatory role in inhibitory control [74] and decision-making behavior in addicts [75]. Based on the previous studies and our results above, we argue that the altered functional connectivity in ACC with cerebellum might be a reflection of the neuro adaptation and reorganization of cerebral functional network caused by BQD. These, of course, need further researches.

In addition, lower connectivity was observed in the BQD group in regions from ACC to the regions of the DMN [76], including mPFC and precuneus, which is part of the posterior parietal cortex. DMN might be the most important component of brain network at resting state [11] while it is a set of brain regions (e.g. ACC, mPFC and precuneus) that are identified as deactivated during demanding cognitive tasks and anti-correlated with the fronto-parietal regions [77–80]. The function of DMN is largely connected with self-relevant, internally directed information processing [79]. Studies of heroin and cocaine dependent individuals have found that hypo activation in the rostral ACC and mPFC is associated with deficits in response inhibition and impulse control [81, 82]. Li indicated that adequate inhibitory control and low craving levels may be caused by an increase of processing in the PFC-ACC, and in states with hypoactivity of the PFC-ACC circuits increased activity of the stress and reward centers, which results in a greater susceptibility to drug use and relapse [82]. Therefore, it can be concluded that disrupting monitoring function in resting-state ACC/mPFC may lead to aberrant behavior in BQD individuals. The functional connectivity of right ACC-left precuneus has a negative correlation with the durations supported the role of precuneus executive control dysfunction in BQD individuals, the BQD may need greater functional connectivity of precuneus to keep executive and inhibitory control balance, but it was further decreased with the durations of BQD. Overall, this study could exhibit the impairment of DMN in BQD individuals from a perspective of functional connectivity.

It was recognized that limbic regions connected with the hypothalamus could also influence addictive behaviors [83]. The lateral hypothalamus was intimately involved in reward, and the hypothalamic peptides, including galanin, enkephalin, orexin and melanin-concentrating hormone, could also stimulate alcohol intake [84–86]. But our outcomes of functional connectivity in regions from ACC to the left para Hippocampal/hypothalamus were decreased, so we argue that might be a reflection of abnormal inhibitory function related to ACC to mesocorticolimbic regions and hypothalamus caused by BQD.

The current study has several limitations. First, the abnormal functional connectivity with ACC in the BQD can only be observed as a cross-sectional study. However, we cannot draw direct causal inferences in terms of the relations between the brain disconnections and BQD. Hence, it would be beneficial to make further longitudinal studies with fMRI experiments in order to set up the cause—effect relations. Second, there was no diagnostic criterion for BQD-related executive dysfunction or disinhibition, and our understanding of the results was limited by such lack of objective and specific neurocognitive assessment. Should such clinical assessment become available, would allow researchers to explore BQD individuals’ brain patterns. More sophisticated clinical assessment (such as Digit Symbol Test, Trail Making Test and the Stroop Color-Word Test to measure cognitive function, Barratt Impulsiveness Scale to compared self-report impulsivity), may provide more accurate interpretation of relative functional connectivity characteristics. Third, given the research indicating that ACC abnormalities play a role in addiction more generally, it is likely that reduced right ACC spontaneous brain activity would also confer risk for other aspects of problematic substance use. Our findings need to be further verified with other drug dependence. Future explorations are still needed to confirm the conclusions.

## Conclusions

Generally, the circuits make interactions and operate simultaneously. However, we found there were significant differences in them between BQD and HC, which is possibly due to the deficiency of reciprocity in these circuits and dysregulation of the integrative systems of rsFC in BQD individuals, and therefore results in maladaptive behaviors. To our understanding, this research is the first rs-fMRI investigation demonstrating an association of BQD behavior with the ACC functional connectivity. Despite those limitations, the abnormal cerebral functional connectivity can also contribute to revealing the neuropathologic mechanisms of BQD. Individuals with BQD develop increased ACC functional connectivity to the areas of the reward network and decreased to the DMN, especially increased functional connectivity in the right ACC- left thalamus and left ACC-pons, may, therefore, represent a biomarker of BQD individuals. Additionally, disturbances in functional connectivity during resting state from ACC to the whole brain revealed by fMRI may have a key function in providing insights into the neurological pathophysiology underlying BQD-associated executive dysfunction and disinhibition. Such results may contribute to our better understanding of the mechanisms of BQD. And further researches and analyses will be carried out for the purpose of detecting the reciprocal functions of each circuit which is affected by BQD.

## Abbreviations

BQD: betel quid dependence
ACC: anterior cingulate cortex
fMRI: functional magnetic resonance imaging
VTA: ventral tegmental area
AN: areca nut
VBM: voxel based morphometry
BOLD: blood oxygen level-dependent
BQDS: BQD Scale
SAS: Self-Rating Anxiety Scale
SDS: Self-Rating Depression Scale
DMN: default mode network
PCC: posterior anterior cingulate
MTG: middle temporal gyri
ITG: inferior temporal gyri
AN: affective network
SMN: sensorimotor network
CN: cognitive network
dlPFC: dorsolateral prefrontal cortex
vlPFC: ventrolateral prefrontal cortex
VN: visual network
mPFC: medial prefrontal cortex
